# On-Surface Modification of Copper Cathodes by Copper(I)-Catalyzed Azide Alkyne Cycloaddition and CO_2_ Reduction in Organic Environments

**DOI:** 10.3389/fchem.2019.00860

**Published:** 2019-12-17

**Authors:** Ryota Igarashi, Ryuji Takeuchi, Kazuyuki Kubo, Tsutomu Mizuta, Shoko Kume

**Affiliations:** Department of Chemistry, Graduate School of Science, Hiroshima University, Higashihiroshima, Japan

**Keywords:** CO_2_ reduction, copper cathode, CuAAC, organic modification, hydrocarbons

## Abstract

In this study, organic structures were introduced onto copper cathodes to induce changes in their electrocatalytic CO_2_ reduction activity. Poorly soluble organic polymers were distributed onto the copper surface as a thin layer by polymerizing monomeric precursors *via* a copper(I)-catalyzed azide-alkyne cycloaddition (CuAAC) activated by anodization of the copper substrate. The resulting structure possesses copper surface atoms that are available to participate in the CO_2_ reduction reaction—comparable to close-contact organic structures—and stabilize the adsorption of organic layers through the CO_2_ reduction process. The CO_2_ reduction performance of the on-surface modified copper cathode exhibited improved CO_2_ reduction over H_2_ evolution compared with traditional cast modification systems. Preventing organic moieties from forming densely packed assemblies on the metal surface appears to be important to promote the CO_2_ reduction process on the copper atoms. The suppression of H_2_ evolution, a high methane/ethylene ratio, and the influence of stirring demonstrate that the improved CO_2_ reduction activity is not only a result of the copper atom reorganization accompanied by repeating anodization for modification; the organic layer also apparently plays an important role in proton transfer and CO_2_ accumulation onto the copper surface.

## Introduction

The reduction of CO_2_ on metal copper cathodes has been of interest since it was first reported by Hori in 1985 (Hori et al., 1985, 1986). The specific character of copper allows highly reduced hydrocarbons to be obtained at relatively large negative potentials, unlike other catalysts, which mainly afford formate and CO (Gattrell et al., 2006; Peterson and Nørskov, 2012; Zhang et al., 2014; Feaster et al., 2017).

Since common polycrystalline copper surfaces can yield various types of hydrocarbons, product distribution is difficult to control, especially for the selective formation of valuable C2-C3 products such as ethylene and ethanol. Product distribution is known to be highly influenced by the applied potential (Hori et al., 2003; Gattrell et al., 2006; Kuhl et al., 2012), Cu crystal facet (Hori et al., 2003; Gupta et al., 2006; Schouten et al., 2012; Huang et al., 2017; Qiu et al., 2017), and proton transfer conditions (Hori et al., 1997; Singh et al., 2016; Varela et al., 2016; Ooka et al., 2017). Oxide-derived Cu nanostructures are of current interest (Kas et al., 2014; Ren et al., 2015; Dutta et al., 2016; Handoko et al., 2016; Mistry et al., 2016; Huang et al., 2017; Mandal et al., 2018); however, their high C2-C3 selectivity has not been fully elucidated, as these materials typically include multiple Cu facets endowing various activities. Furthermore, their nano-scale morphology influences local pH, and the remaining oxygen atoms are considered to influence the electronic nature of the surface. Other nanostructured Cu materials synthesized *via* various preparation methods (Tang et al., 2011; Li and Kanan, 2012; Reske et al., 2014; Ma et al., 2015; Kim et al., 2017; Zhao et al., 2017; Jeon et al., 2018; Luna et al., 2018) and alloys comprising copper and other elements are reported to show excellent performance in C2-C3 product formation (Long et al., 2017; Ma et al., 2017; Zhang et al., 2017).

The introduction of organic structures onto catalytically active metal surfaces has recently received attention, particularly the preparation of self-assembled monolayers (SAMs). Traditionally, the adsorption of organic molecules has been used to deactivate pristine metal surfaces and is often exploited to achieve higher selectivity, as demonstrated by the Lindlar catalyst. Recent developments have shown that organic molecules have a positive impact on enhancing selectivity and activity (Schoenbaum et al., 2014). Regarding CO_2_ reduction, several groups have introduced organic molecules onto copper surfaces as SAMs to improve the reaction selectivity of CO and CO_2_ reduction (Xie et al., 2016; Gong et al., 2017; Ahn et al., 2018). In these studies, phase-separated and densely packed structures of organic molecules on metal surfaces are carefully avoided, and the design of open-surface metal centers with neighboring organic structures to allow their cooperation seems to be a prerequisite for a productive reaction environment. To obtain the right reaction environment, similar to coordination catalysts, in which vacant metal centers can cooperate with organic ligands, requires that specific strategies be adopted in relation to contact-surface preparation.

**Figure 1:**
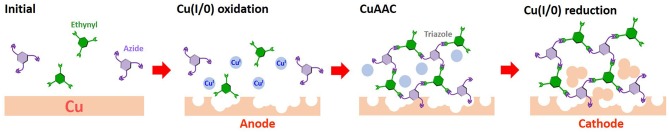
On-surface CuAAC modification scheme.

Herein, we have developed a new method to modify metallic copper cathodes with organic layers, in contrast to SAM adsorption routes. To place open metal surface atoms in the neighborhood of the organic structure and to enhance stability, we adopted a rigid organic polymer structure. These polymers are poorly soluble in common solvents and are difficult to distribute homogeneously on the surface without aggregation. To distribute the polymer structure across the surface as a thin layer, the monomeric precursors were polymerized on the copper electrode by exploiting the Cu(I)-catalyzed azide-alkyne cycloaddition (CuAAC) catalytic activity response to surface oxidation (Figure 1). In this report, the copper modification achieved *via* the on-surface CuAAC approach was studied, and an efficient CO_2_ reduction activity, specific to the polymer-modified electrode, was observed.

## Layer Preparation and Characterization

### Preparation

**Figure 2:**
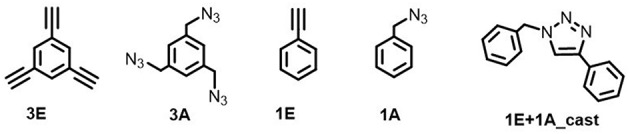
Ethynyl and azide precursors.

**Figure 3:**
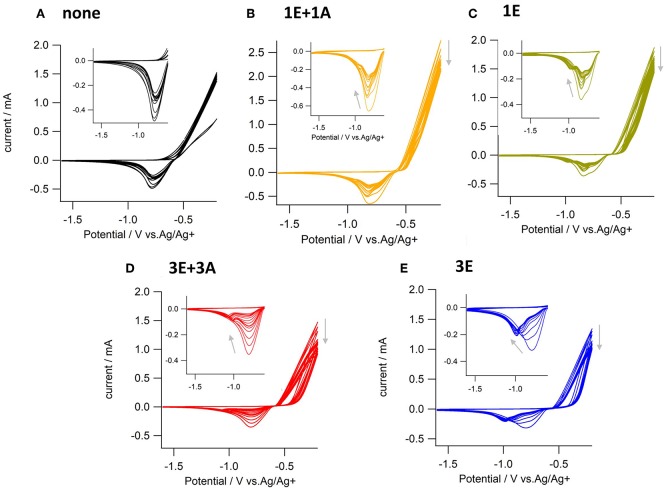
Cyclic voltammograms of copper electrodes scanned 15 times in electrolyte solution containing **(A)** no additives, **(B) 1E** and **1A, (C) 1E, (D) 3E** and **3A**, and **(E) 3E**. Electrolyte solution; 0.1 M ^n^Bu_4_NPF_6_-acetonitrile, scan rate; 0.1 Vs^−1^.

Electrochemical on-surface modification was performed in an electrolyte solution containing ethynyl and azide monomers (Figure 2). The redox activity of the surface copper was observed by repetitive anodic scanning of the polycrystalline copper electrode. In the absence of organic additives, the dissolution of oxidized copper from the surface proceeded continuously with an onset potential of *ca*. −0.6 V (Figure 3A). The first anodic scan exposed a fresh surface, making the current double afterward, and the anodic current profile was constant without decrease in the subsequent scans. When **1E** or **3E** was present in the solution, the onset potential of the copper anodization shifted positively, indicating that the ethynyl moieties promoted adsorption on the copper surface and thus inhibited copper stripping (Figures 3B–E). The appearance of a negatively shifted cathodic peak at *ca*. −1 V indicates coordination of the dissolved copper ion species. The ethynyl moiety likely formed insoluble coordination polymers with copper(I) through sigma- and pi-coordination bonds (Abrantes et al., 1984), and the anodic scanning appeared to first dissolve the copper(I) species into the solution (Ahrland, 1982), with subsequent coordination of ethynyl moieties. When azide precursors coexisted, these peaks were less recognizable (Figures 3B,D), implying that ethynylcopper(I) was consumed in the subsequent CuAAC reaction. Upon scanning, the anodic current gradually decreased, and an anti-corrosive growth layer was observed on the surface. The current decrease was prominent in **3E+3A**, and to a smaller degree, when the solution contained **3E**, **1E** alone or **1E+1A**. The covalent polymers composed of **3E+3A**, via the CuAAC reaction, appear to induce strong adsorption compared with **3E** without covalent bond formation. The anodic scanning of **1E+1A** is expected to afford monomeric 1,2,3-triazoles because they contain only one reaction point for each, resulting in very weak adsorption. These features show that the anticorrosive layer efficiently covered the Cu surface by anodization with **3E+3A**.

### X-Ray Photoelectron Spectroscopy (XPS)

**Figure 4:**
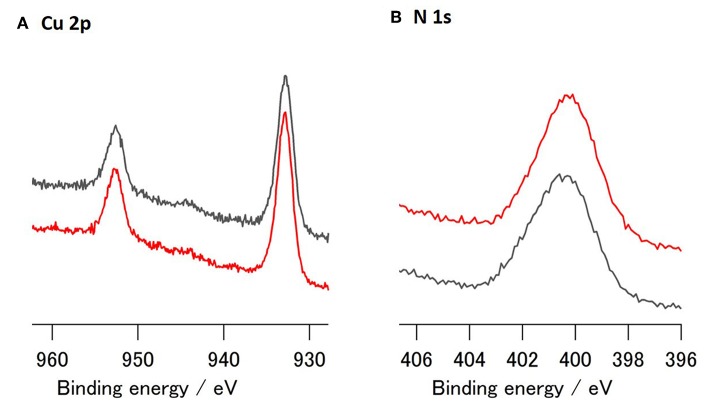
XPS spectra of modified Cu; **(A)** Cu2p and **(B)** N1s region of **Cu_3E+3A** before (red) and after (gray) CO_2_ reduction (−1.4 V vs. RHE, 40 min).

XPS analysis of the **Cu_3E+3A**-modified Cu surface showed that a fair amount of nitrogen was introduced onto the surface (Figure 4). The binding energy associated with the introduced N species does not indicate an azide moiety, as this exhibited a peak located at ~405 eV (Collman et al., 2006; Chisholm et al., 2016). The majority of the nitrogen, when affixed to the surface, appeared in the form of a triazole. Conversely, in addition to the Cu 2p peaks, **Cu_1E+1A** also exhibited a small nitrogen peak upon modification (Figure S1). These nitrogen features correspond to the difference in adsorption of polymeric and monomeric structures, as discussed in the on-surface preparation section, showing a significant and robust organic modification in polymeric **Cu_3E+3A**.

### Scanning Electron Microscopy (SEM)

**Figure 5:**
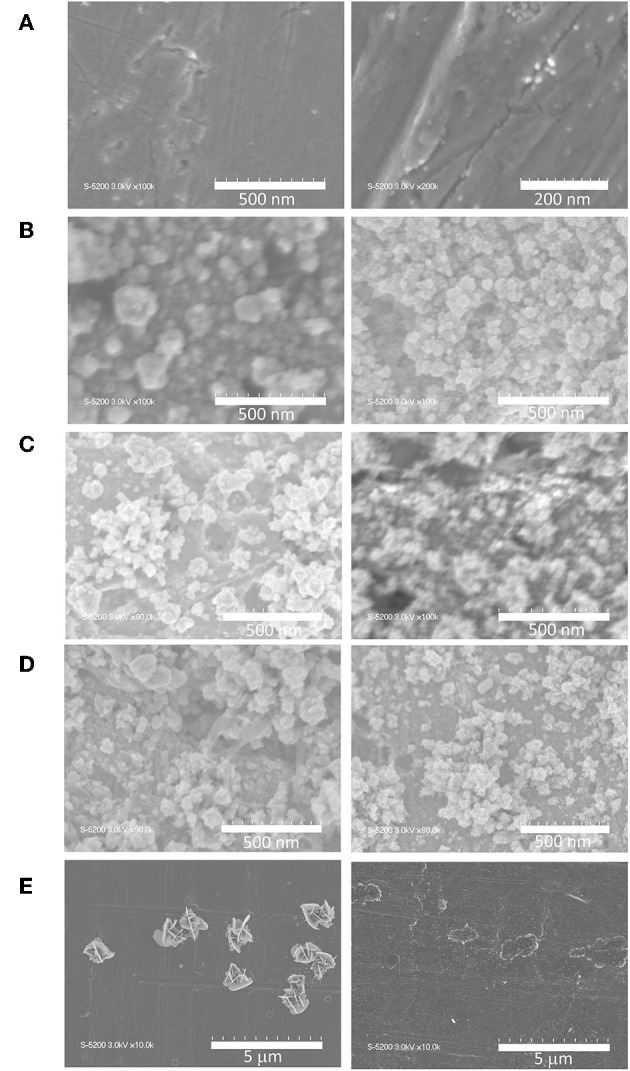
SEM images of copper electrodes; (left) as prepared and (right) after electrolysis for 40 min at −1.4 V vs. RHE, 0.1 M KHCO_3_aq saturated with CO_2_. **(A) Unmodified, (B) Cu_3E+3A, (C) Cu_1E+1A, (D) Cu_1E**, and **(E) Cu_1E+1A_cast**.

During the on-surface preparation, a significant amount of grain structures grew from an initially smooth polished Cu polycrystal surface (Figure 5). Additionally, the structures of **Cu_3E+3A**, **Cu_1E+1A**, and **Cu_1E** were similar. Anodic scans of the copper surface result in dissolution and re-deposition of Cu^+^ in acetonitrile in the presence of phenylacetylene, and insoluble copper phenylacetylide is introduced as a stable intermediate (Ahrland, 1982). The grain structures seemed to grow through an oxidation–coordination–reduction process in repetitive anodization cycles, similar to the preparation of copper nanostructures derived from copper oxide CO_2_-reduction catalysts. When the number of scan cycles was reduced to seven, the surface was covered by a smaller structure in the early stage of growth (Figure S2). XPS showed that **Cu_3E+3A** contained a higher amount of organic groups on its surface than **Cu_1E+1A**. However, these structures had very similar appearances, implying that the organic moieties were uniformly dispersed on copper grains. The thickness of the rough surface structure is estimated to have been hundreds of nanometers from the cross-sectional SEM image (Figure S3). When molecular triazole was cast on the copper surface (Figure 5E), platelet crystals were formed on the surface. Additionally, the basal copper itself was observed to be slightly roughened by casting (Figure S4), as discussed in the roughness factor section.

### Roughness Factor

The roughness of the electrodes was calculated from the double-layer capacitance (Figure S5, Table S1). Table 1 details the roughness factors of the Cu electrodes, with the unmodified electrode as the reference. **Cu_1E+1A** and **Cu_1E** exhibited a high degree of roughness, which is consistent with the apparent surface topology observed in the SEM micrographs. Despite the apparent granular morphology, the roughness of **Cu_3E+3A** was similar to that of the smooth unmodified electrode. The surface of the Cu electrode may have been partially insulated by the presence of a thick organic layer film. When the triazole molecule was cast onto the surface, the surface area slightly increased, although no anodization of the copper was achieved. The adsorption of densely packed triazole, and the subsequent cathodization, may result in the re-construction of the surface copper (Gunathunge et al., 2017), as observed in the SEM micrographs.

**Table 1:** Roughness factors of copper electrodes.

	**Unmodified**	**Cu_3E+3A**	**Cu_1E+1A**	**Cu_1E**	**Cu_1E+1A_cast**
	1.0	1.8	4.0	3.7	1.4
After EL^a^	0.9	1.9	4.3	3.9	1.7

a *Electrolysis was carried out for 50 min from −1.0 to −1.4 V vs. RHE with a stepwise increment of −0.1 V in every 10 min*.

### Underpotential Deposition (UPD)

**Figure 6:**
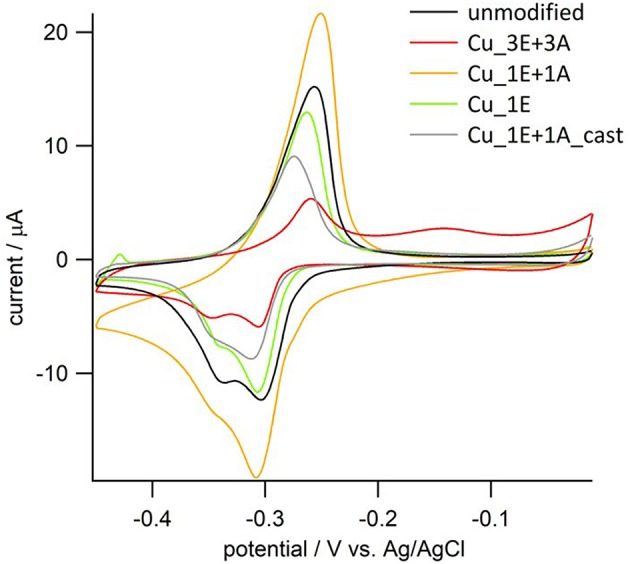
Cyclic voltammograms of copper electrodes in the solution containing 0.5 mM PbCl_2_. Electrolyte; 0.5 mM HCl/0.01 M HClO_4_aq, scan rate; 10 mVs^−1^.

The amount of exposed Cu atoms on the electrode surface was estimated by UPD analysis (Figure 6). There were fewer exposed Cu atoms in the **Cu_3E+3A** electrode than estimated on the basis of the SEM micrograph and roughness factor. The surface of **Cu_3E+3A** is considered to have been largely covered by the organic layer, which inhibited the approach and deposition of Pb^2+^; however, the system maintained its function as an electrode, as the layer could almost be considered as a monolayer in terms of thickness (Bandyopadhyay et al., 1998; Feng et al., 2017). The size of the redox wave was significantly larger in **Cu_1E+1A**, as the monomeric triazole was not able to strongly adsorb onto the surface. Furthermore, the majority of the grain structure was composed of copper. In **Cu_1E+1A_cast**, the organic moieties seemed to be aggregated to form massive crystals that inhibited the Pb deposition process on the electrode surface.

## CO_2_ Reduction

**Figure 7:**
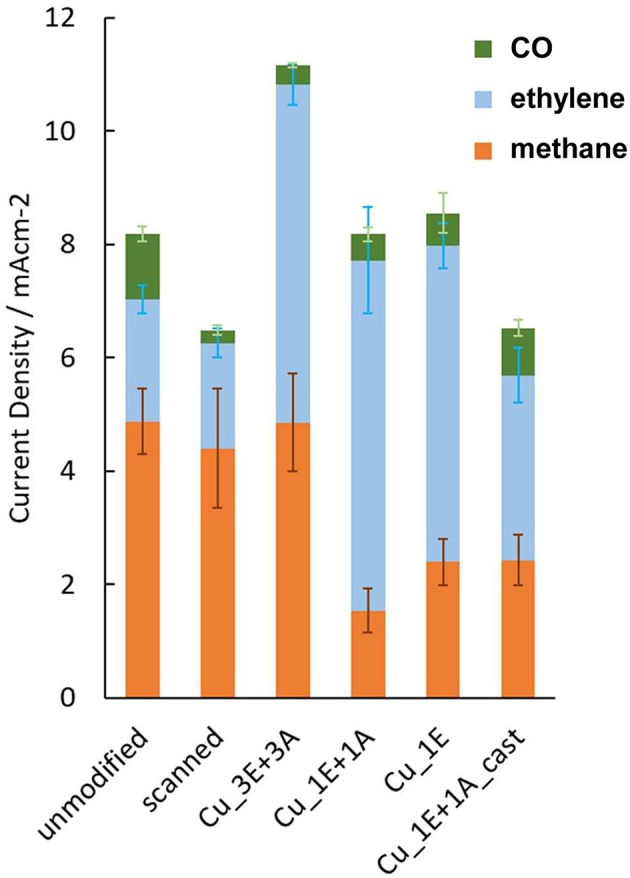
The partial current density for the gas-phase carbonaceous products from electrolysis at −1.4 V vs. RHE; Electrolyte; CO_2_-saturated 0.1 M KHCO_3_. Error bars are placed at the top of each component. The scanned electrode was prepared by the same anodic scanning process as for on-surface preparation, without the addition of ethynyl or azide precursors.

The formation of carbon products by CO_2_ reduction at −1.4 V (vs. reversible hydrogen electrode, RHE, solution resistance uncorrected, Figure 7) showed that **Cu_3E+3A** had the highest amount of carbonaceous product formation. The products demonstrate two remarkable effects of modification: (i) ethylene formation was enhanced by anodic scanning in the presence of the ethynyl precursors; (ii) only the **Cu_3E+3A** electrode exhibited improved methane formation as a result of modification, while the other electrodes appeared to compensate for ethylene formation with methane formation.

**Figure 8:**
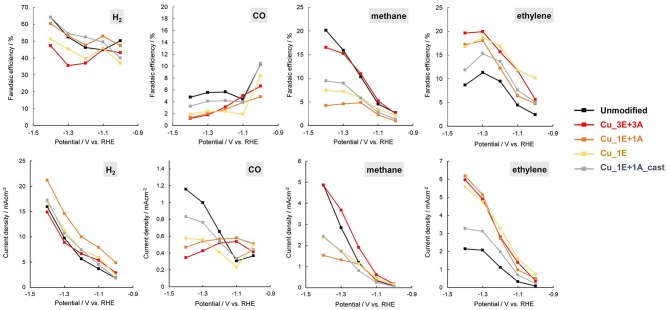
Potential dependence of current density Faradaic efficiency **(top)** and current density **(bottom)** for product of CO_2_ reduction.

Comparison of the Faradaic efficiency (Figure 8) reveals that the high CO_2_ reduction performance of **Cu_3E+3A** did not result simply from an increase in total current with increasing roughness of the electrode surface. Besides the increase in methane and ethylene formation, **Cu_3E+3A** exhibited remarkably low hydrogen production, which is undesirable for the proton-consuming side reaction. Also, the control experiment under argon only bore hydrogen from **Cu_3E+3A** at the potential range, showing that the modified organics were not the source of hydrocarbons (Figure S6).

**Figure 9:**
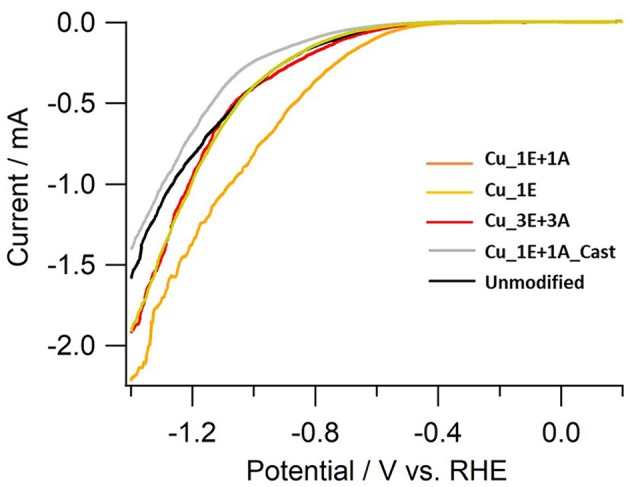
LSV of copper electrodes. Electrolyte; CO_2_-saturated 0.1 M KHCO_3_, scan rate; 20 mVs^−1^.

The linear sweep voltammetry (LSV) curves (Figure 9) of the electrodes provide additional information on reaction selectivity. At low current densities (−0.4 to −0.8 V), the current ratio appears to largely depend on the roughness factor. Further reducing the negative potential to under −1.0 V resulted in similar current densities for all electrodes (*ca*. −2 mAcm^−2^), despite observed differences in the roughness factor. At high current densities, the thickness of the CO_2_ and proton diffusion layer increased beyond the scale of the thickness of the deposited surface structures. Therefore, the reaction is limited by the transportation properties. The polarization involves multiple electrochemical processes. By incrementally increasing the potential, the onset of CO_2_ reduction to CO and hydrogen evolution was observed to first emerge at approx. −0.4 V, and the reduction of adsorbed CO into hydrocarbons was further enhanced when the potential was under −1.0 V. The current in **Cu_3E+3A** displayed slow increments despite of a relatively positive current onset, indicating that significant CO adsorption results in a current drop until the potential reaches −1.0 V, as CO_2_ reduction is significantly more efficient than H_2_ evolution. Thereafter, the current rapidly increased below −1.0 V as the adsorbed CO was removed through further reduction into hydrocarbons followed by dissociation, consistent with the potential dependence of Faradaic efficiency for H_2_ and CO (Figure 8).

**Figure 10:**
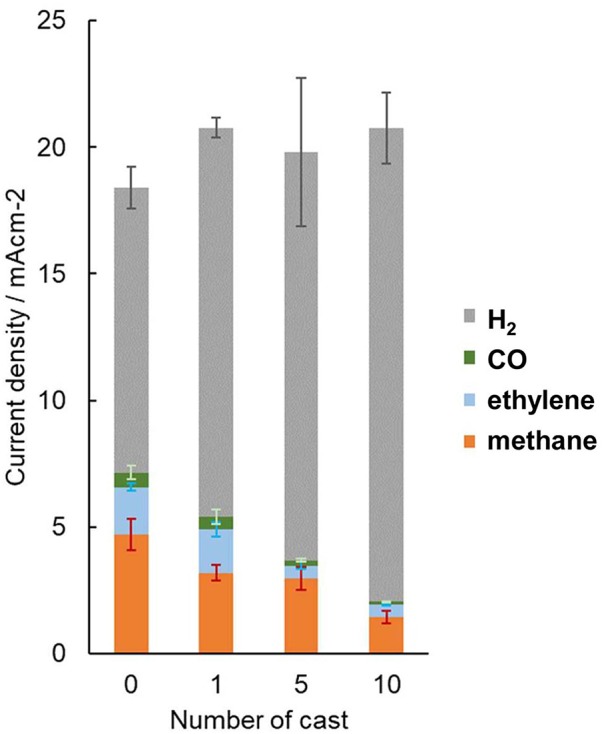
Dependence of partial current density of the gaseous product from cast-modified electrodes with the number cast processes. Error bars are placed at the top of each component.

The effect on CO_2_ reduction is influenced by how the organic moieties contact with the copper surface. In repeated cast modifications of the polished Cu electrode, the total current density was not observed to decrease; however, the formation of carbonaceous products decreased depending on the number of times the copper electrode was subjected to the cast process and was even surpassed by hydrogen production (Figure 10). The casting of molecular triazole typically forms densely packed crystalline structures that inhibit the adsorption of CO_2_ on the surface. Conversely, this is not the case for H_2_ production, as H_2_ evolution requires significantly smaller surface vacancies to adsorb H atoms, and it may even be possible on the adsorbed organic molecules.

An ethylene increase was observed for the electrodes that were subjected to repetitive anodic scanning in the presence of the ethynyl precursor. The existence of the insoluble copper(I) acetylide intermediate on the surface is important for copper reorganization. This observed trend is similar to the high ethylene formation efficiency observed for nanostructured CO_2_-reduction catalysts that are prepared by the formation of an insoluble copper oxide layer on the surface followed by reduction to Cu(0) (Kas et al., 2014; Ren et al., 2015; Dutta et al., 2016; Handoko et al., 2016; Mistry et al., 2016; Mandal et al., 2018). During anodization in the absence of the ethynyl precursor (“scanned” in Figure 7), CO_2_ reduction resulted in a decrease in the efficiency of ethylene formation. Continuous dissolution of copper into acetonitrile, without the precursor, may remove the copper atoms at the defect site of the copper crystal facet.

In the majority of Cu electrocatalytic processes, there is a trade-off in the efficiency of methane and ethylene selectivity. Additionally, the pH of the electrolyte influences the ratio of methane and ethylene, as the reduction process is related to competition of the dimerization of the adsorbed CO and the proton-coupled reduction (Schouten et al., 2011, 2013; Huang et al., 2017). The potential dependence of the ethylene/methane ratio also shows that methane formation is dominant with increased current density, as the reduction rate surpasses CO dimerization. Further increasing the current density results in an increase in pH on the surface due to H^+^ consumption, resulting in dominant H_2_ production through H_2_O reduction because CO_2_ is not stable in alkaline media (Singh et al., 2016). This trend appears to hold true in the case of **Cu_1E+1A** and **Cu_1E**, as the total CO_2_ reduction efficiency remained at a similar level to that of the unmodified electrode. In **Cu_3E+3A**, both methane and ethylene increased with remarkably low hydrogen production. Hence, the organic layers play a role in increasing the total CO_2_ reduction efficiency.

**Figure 11:**
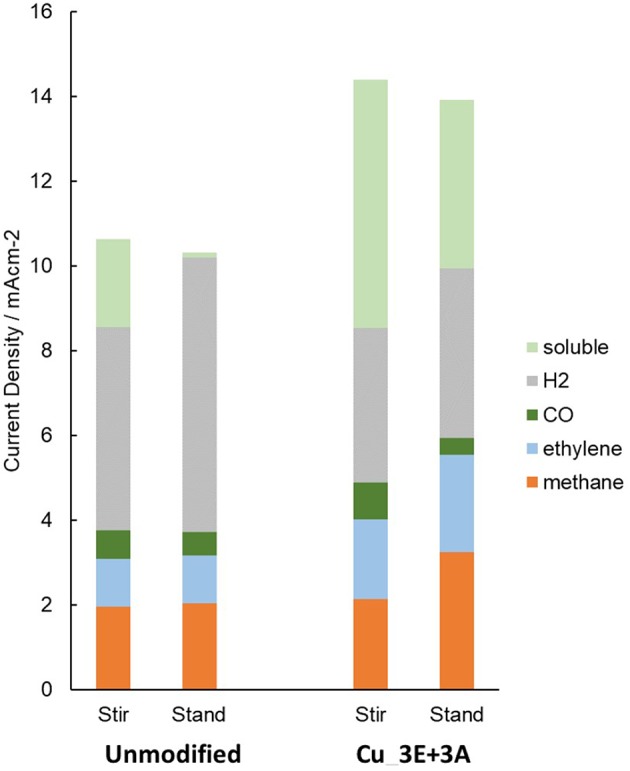
Partial current density of the reduction products. The current density of the soluble product was estimated from the remaining component of the total current density after subtracting the gaseous components.

Stirring the electrolyte solution also changes the transport conditions. When stirring of the solution was stopped (Figure 11), the amount of dissolved components in the electrolyte (most likely formate) decreased for both the unmodified and **Cu_3E+3A** electrodes. Hydrogen was observed to increase for the unmodified electrode, but with the **Cu_3E+3A** electrode, the decrease of the soluble product content appeared to be compensated for by hydrocarbon formation, especially methane. Under a high current-flow condition without stirring, a pH increase at the electrode surface was observed to occur, as every electron transfer was accompanied by a proton transfer. Furthermore, the depletion of CO_2_ was more severe for two reasons: (i) CO_2_ possesses a lower diffusion coefficient than H^+^, and (ii) CO_2_ transforms to ${\text{HCO}}_{3}^{-}$ in basic media. The suppression of hydrogen evolution by modification of the electrode appears to result from the preference of CO_2_ reduction and adsorption over the reduction of H_2_O. CO_2_ selectivity is affected by the competition between CO_2_ reduction and proton reduction forming surface-adsorbed H, and the latter process tends to lead to the formation of H_2_ and formate. Several recent reports have demonstrated that hydrophobic modification of the surface improves the supply of CO_2_ relative to H^+^, which leads to better selectivity to CO and hydrocarbons (Buckley et al., 2019; Li et al., 2019). Moreover, the three-way triazole moiety was reported to have specific affinity in MOF CO_2_ storage (Wang et al., 2013). In our case, the introduction of the organic layer made the surface more hydrophobic (Figure S7). The presence of the organic layer seems to create a CO_2_-rich environment on the electrode surface, possibly by improved proton transfer and better CO_2_ affinity with the layer than with the bare electrode surface.

## Conclusions

A novel method has been designed to modify the surface of a metal electrode to enhance surface catalytic activity by anodization. The metal-organic contact structure formed offers unique properties over those of traditional cathodes prepared *via* the cast method by inducing a thin layer of organic moieties that are accessible to surface copper atoms. The influence of the modified electrode on CO_2_ reduction reveals that the contact surface has a preference toward CO_2_ reduction over H_2_ evolution, contrary to the observed results from surfaces modified by the cast method. The organic layer appears to enhance the environment to achieve improved CO_2_ affinity and proton transport ability at the surfaces of the copper atoms. The re-organization of copper atoms upon modification did influence CO_2_ reduction selectivity, as in copper oxide-derived electrocatalysis. However, this property is not the sole factor governing the observed CO_2_ reduction selectivity, because a large total CO_2_ reduction efficiency and high methane formation are observed, even at low potentials. The method herein can readily introduce various types of organic substituents to the organic-open metal contact surface. Studies are underway to investigate controlling the CO_2_ reduction based on the discussion in this report. Furthermore, the introduction of an organic functional center, such as ligands and dyes, would allow for CO_2_ reduction with cooperated activation or light-driven functionality.

## Methods

### Electrode Modification

A disc copper electrode (3 mmφ) was made from the cross-section of a polycrystalline rod embedded in a glass tube and polished with Bicalox0.05CR (0.05 μm alumina abrasive). Modification was performed in 5 mL electrolyte (0.1 M ^n^Bu_4_NPF_6_-acetonitrile) containing ethynyl (6.6 mM) and azide (6.6 mM) precursors under an argon atmosphere. A potential scan was repeated 15 times (from −0.2 to −1.0 V vs. Ag/Ag^+^, 0.1 Vs^−1^) using an ALS 650D potentiostat, followed by washing with acetonitrile. For cast modification, 0.7 μL of acetonitrile solution containing 1-benzyl-4-phenyl-1,2,3-triazole (4.3 mM) was dropped on the copper disc and air-dried. Modified copper substrates for XPS and SEM measurements were prepared using 5 mm square copper foil instead of copper discs.

### Characterization of Surface Structure

SEM images were collected with a Hitachi FE-SEM S-5200. XPS data were collected using a KRATOS ESCA 3400 with an Al-*K*α source. Double-layer capacitance was measured in CO_2_ saturated 0.1M KHCO_3_aq on the basis of the current dependence upon the potential scan rate in the region of −0.6 V to −0.7 V vs. Ag/AgCl. UPD was achieved by immersing the modified electrode in electrolyte-containing lead salt (0.5 mM PbCl_2_, 0.5 mM HCl/10 mM HClO_4_aq, scan rate; 10 mVs^−1^).

### CO_2_ Reduction

The CO_2_ reduction activity on copper electrodes was investigated in CO_2_-saturated 0.1 M KHCO_3_aq (pH 6.8). The three-electrode setup was connected to a potentiostat (ALS 650D). Ag/AgCl was used as a reference electrode, and Pt mesh was used as the counter electrode. The applied potential was converted to RHE according to the equation *E*_RHE_ = *E*_Ag/AgCl_ + 0.197 V + 0.0591 × pH.

The experiments were performed in a two-compartment cell. A copper working electrode and Ag/AgCl electrode were placed in the gas-tight cathodic compartment. It was separated from the open anodic compartment, which was equipped with a Pt mesh counter electrode, by a 10 mmφ ion-exchange membrane (Selemion^TM^ AMV). Electrolysis was performed for 10 min with stirring (ca. 600 rpm), without additional supply of CO_2_. Depletion of CO_2_ by electrolysis did not exceed a few % of the amount of CO_2_ in solution (34 mM, 6.2 mL), which is estimated from the charge flow (typically 1 C). For the quantification of gaseous products, 0.1 mL of gas product was collected from the head space (9.9 mL) of the gas-tight compartment and introduced in a gas chromatograph (Shimazu-2010) equipped with a 2.0 m × 1.0 mm ID column packed with SHINCARBON ST and a BID-2010 detector. Copper electrodes were first cathodized at −1.6 V vs. Ag/AgCl for 1 h to remove weakly physisorbed materials and reduce oxidized copper species prior to the collection of CO_2_RR products. The current density was calculated according to the geometric area of the Cu electrode (3 mm φ). CO_2_ reduction data are provided as averages from at least three separately prepared electrodes.

## Author Contributions

SK, KK, and TM contributed the overall concept of the study. SK and RT developed the experimental apparatus for electrolysis. RT and RI performed the experiments and analysis of the collected data. All authors contributed to manuscript revision and read and approved the submitted version.

### Conflict of Interest

The authors declare that the research was conducted in the absence of any commercial or financial relationships that could be construed as a potential conflict of interest.
