# Supplementary material for: On-Surface Modification of Copper Cathodes by Copper(I)-Catalyzed Azide Alkyne Cycloaddition and CO2 Reduction in Organic Environments
Source: Front Chem. 2019 Dec 17;7:860. doi: 10.3389/fchem.2019.00860 (PMC6951422; doi:10.3389/fchem.2019.00860)
Supplement: Supplementary file 1 [file Data_Sheet_1.pdf]

Supplementary Material

On-surface Modification of Copper Cathodes by Copper(I)-catalyzed Azide Alkyne Cycloaddition and CO_2 Reduction in Organic Environments

Ryota Igarashi, Ryuji Takeuchi, Kazuyuki Kubo, Tsutomu Mizuta and Shoko Kume*

Department of Chemistry, Graduate School of Science, Hiroshima University

Materials

Copper rods (3mmf 99.9%) and Copper foils (0.1mm thickness 99.96 %) were purchased from Nilaco Co. Acetonitrile was distilled from P_2O_5 and stored under nitrogen. $KHCO_3$ was purchased from Katayama Chemicals. CO_2 gas(99.995 %) was purchased from NIPPON EKITAN Co. $^nBu_4NPF_6$ and perchloric acid were purchased from Wako pure chemicals. Ethynylbenzene were purchased from TCI. 1A,[1] 3A,[1.2] 3E[3] and 1-benzyl-4-phenyl-1,2,3-triazole[4] were synthesized according to the literature.

[1] Alvarez, Salvador G.et al., Synthesis, (4), 413-414; 1997
[2] Vonhoeren, Benjamin et al., ACS Applied Materials & Interfaces, 7(13), 7049-7053; 2015
[3] Bayon, Carlos et al., Chemistry - A European Journal, 23(7), 1623-1633; 2017
[4] Lewis, Warren G. et al. Journal of the American Chemical Society, 126(30), 9152-9153; 2004

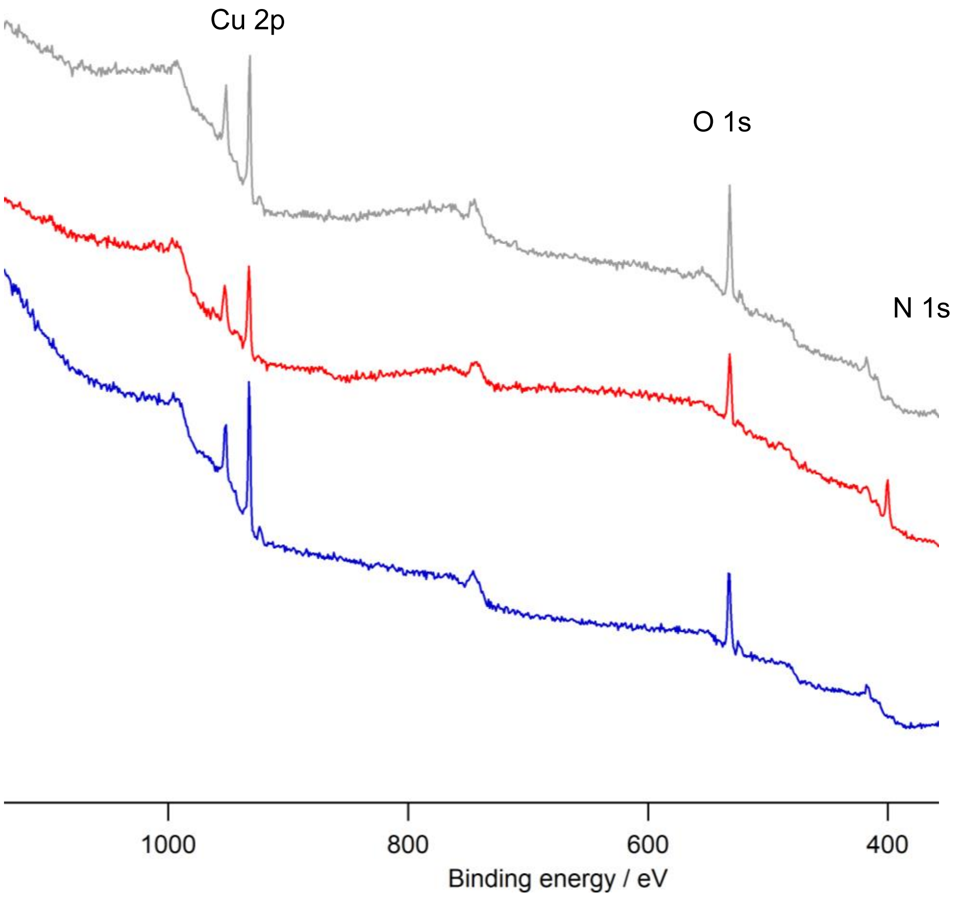

Figure S1 XPS spectrum of **unmodified**(gray), **Cu_1E+1A**(blue) and **Cu_3E+3A**(red).

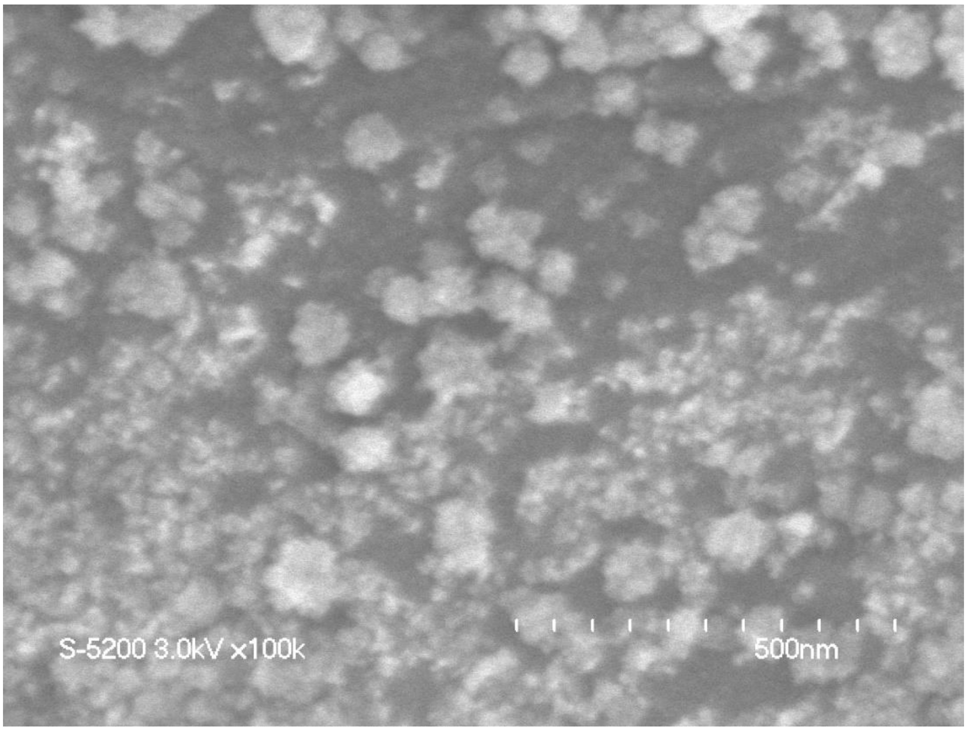

Figure S2 A SEM image of a copper electrode scanned with 7 times in the electrolyte solution contains 3E(6.6 mM) and 3A(6.6 mM). Electrolyte solution; 0.1M $^{n}Bu_4NPF_6$-acetonitrile, scan rate; 0.1 Vs^{-1}.

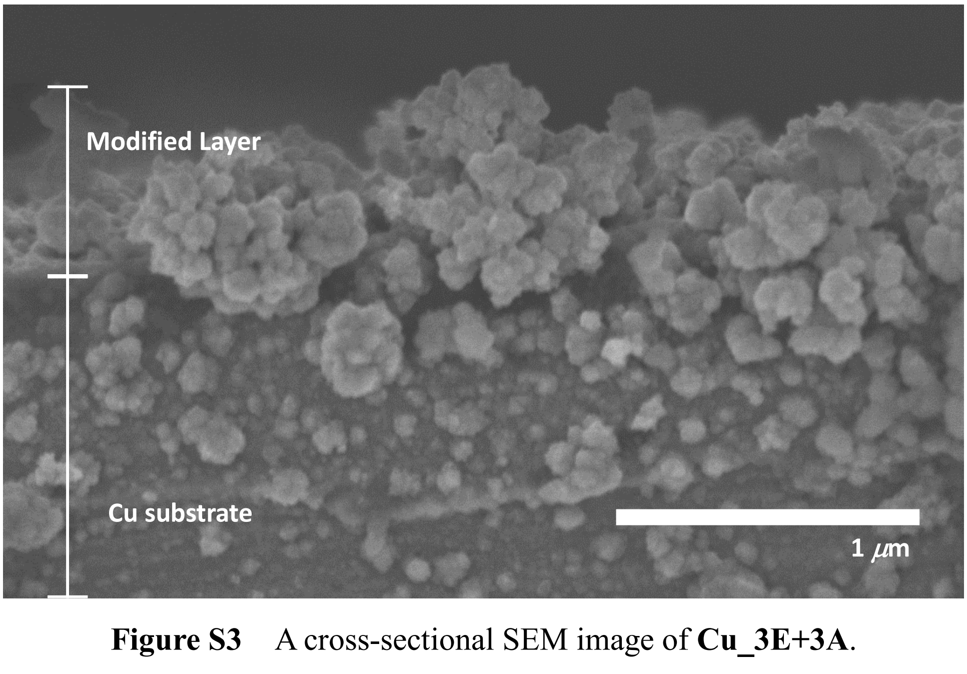

Figure S3 A cross-sectional SEM image of **Cu_3E+3A**.

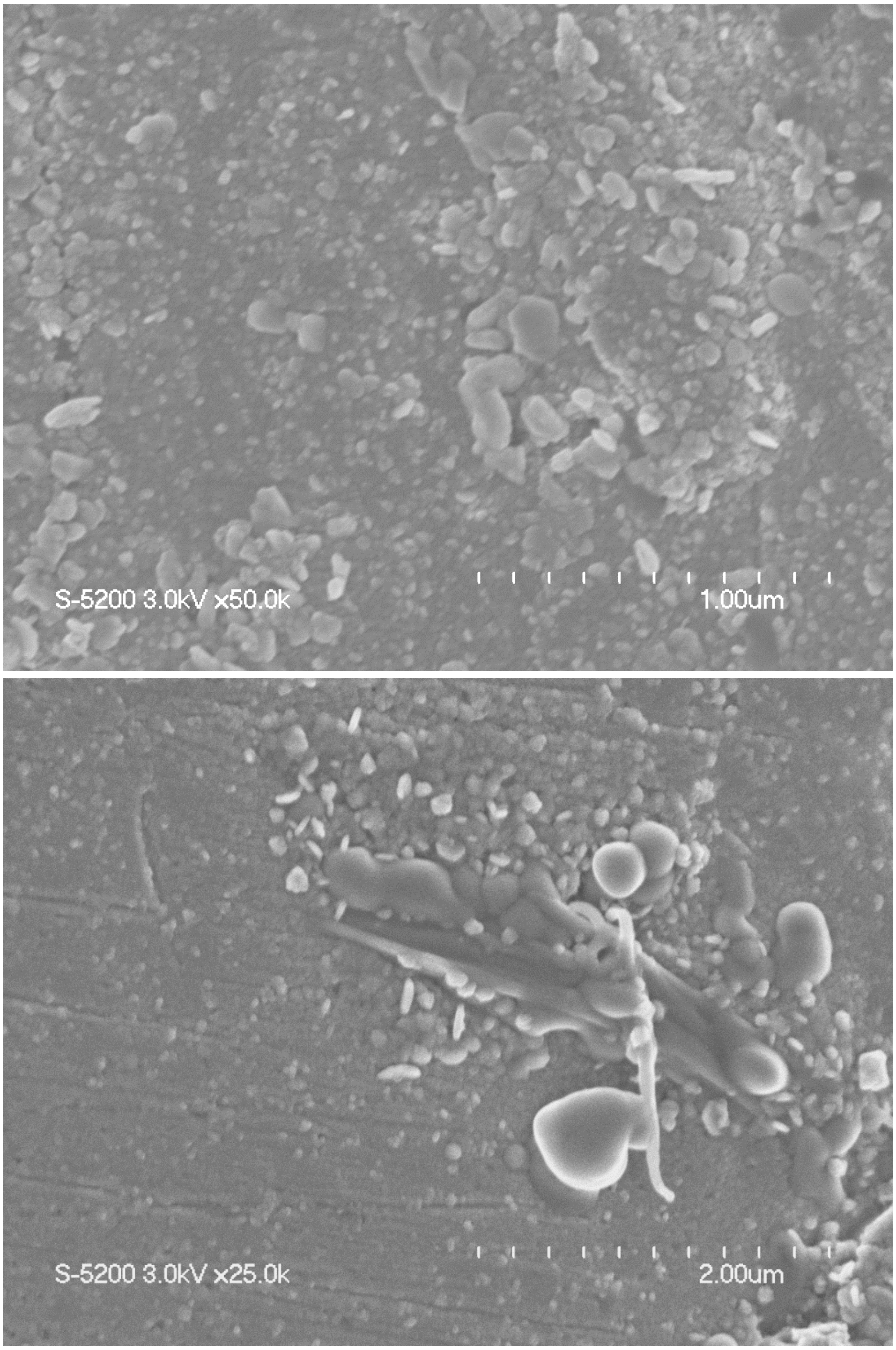

Figure S4 SEM images of **Cu_1E+1A_cast** electrode; (upper) as prepared and (bottom) after electrolysis for 40min at -1.4 V vs.RHE, 0.1 M $KHCO_3$aq saturated with CO_2.

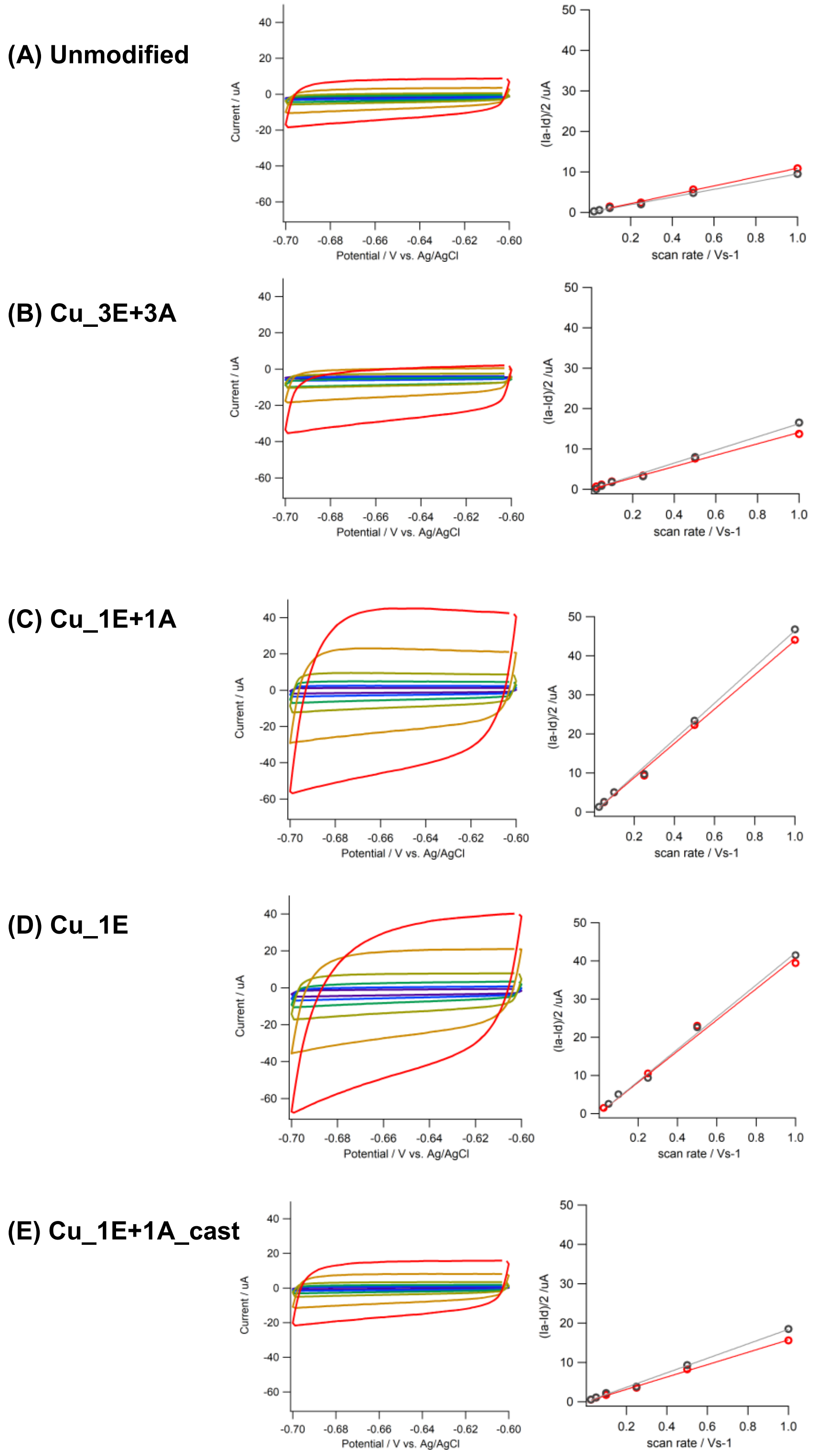

Figure S5 (Left) Cyclic voltammograms of Cu electrodes before CO_2 electrolysis at the region of non-Faradaic process. (Right) Scan rate dependence of current at -0.65 V. red, before CO_2 electrolysis; gray, after CO_2 electrolysis.

Table S1 Double Layer Capacitance of Copper Electrodes (μFcm^{-2})

	**unmodified**	**Cu_3E+3A**	**Cu_1E+1A**	**Cu_1E**	**Cu_1E+1A_cast**
	156	283	627	582	224
After EL[a]	136	301	663	601	263

a) Electrolysis were operated for 50 min from -1.0 V to -1.4 V vs. RHE, with stepwise increment of -0.1V in every 10 min.

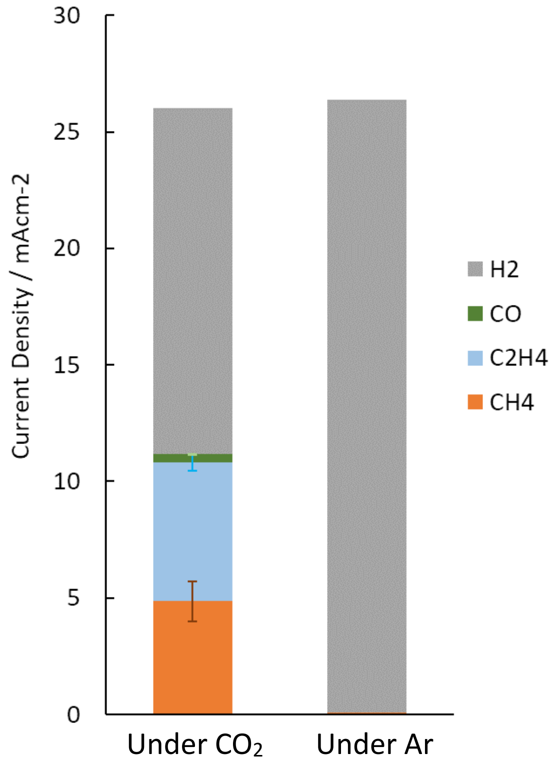

Figure S6 The partial current density for the carbonaceous product in gas phase, by electrolysis on **Cu_3E+3A** at -1.4 V vs. RHE; Electrolyte; CO_2-saturated 0.1 M $KHCO_3$.

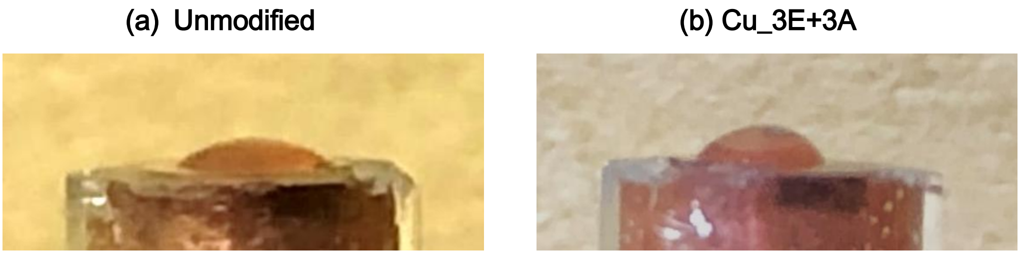

Figure S7 1 μL water droplet placed on the Cu electrodes.
